# The Effects of Boron Neutron Capture Therapy on the Lungs in Recurrent Breast Cancer Treatment

**DOI:** 10.7759/cureus.57417

**Published:** 2024-04-01

**Authors:** Hiromasa Kurosaki, Keita Okazaki, Mihiro Takemori, Etsuko Tate, Tatsuya Nakamura

**Affiliations:** 1 Department of Radiology and Radiation Oncology, Edogawa Hospital, Tokyo, JPN

**Keywords:** radiation pneumonia, first report, breast cancer research, tumor recurrence, boron neutron capture therapy (bnct)

## Abstract

Boron neutron capture therapy (BNCT) has predominantly been performed for brain tumors or head and neck cancers. Although BNCT is known to be applicable to breast cancer, it has only been performed in a few cases involving thoracic region irradiation with reactor-based BNCT systems. Thus, there are very few reports on the effects of BNCT on the thoracic region and no reports of BNCT for breast cancer with accelerator-based BNCT systems. This paper introduces the world’s first clinical study employing an accelerator-based BNCT system targeting recurrent breast cancer after radiation therapy. We aim to assess the efficacy and safety of BNCT, focusing on the dose response in the thoracic region, especially concerning the potential for radiation pneumonitis. Preliminary findings from the first three cases indicate no evidence of radiation pneumonitis within three months post treatment. This study not only establishes a foundation for novel breast cancer treatment options but also contributes significantly to the field of BNCT in the thoracic region.

## Introduction

Boron neutron capture therapy (BNCT) has been developed and performed using nuclear reactors in Japan. However, reactor-based BNCT (RB-BNCT) is not suitable for installation in a hospital since it is costly to build and maintain a nuclear reactor with strict regulations. Consequently, BNCT systems have shifted from RB-BNCT to accelerator-based BNCT (AB-BNCT).

AB-BNCT employs beryllium or lithium targets to produce neutrons for BNCT treatment. The NeuCure® System (Sumitomo Heavy Industries, Ltd., Tokyo, Japan) utilizes a beryllium target and was installed at the Kansai BNCT Medical Center of Osaka Medical and Pharmaceutical University and Southern Tohoku BNCT Research Center. The system has primarily been utilized for the treatment of head and neck cancer. Moreover, BNCT has been approved as insurance coverage for unresectable, locally advanced, and recurring cancer of the head and neck region in 2020.

Conversely, Cancer Intelligence Care Systems, Inc. (CICS). has developed a lithium target system for BNCT, and the AB-BNCT system was installed at the National Cancer Center Hospital (NCCH) in Japan, referred as to CICS-1. Clinical trials for angiosarcoma and melanoma have been performed [1,2]. The same type of accelerator system at the NCCH was later installed at Edogawa Hospital, referred as to CICS-2. We have started a clinical study targeting recurrent breast cancer post-radiotherapy since July 2023. This is a single-center, open-label study, registered as jRCTs031220371 at Edogawa Hospital [3].

This study represents the world's first treatment of breast cancer using AB-BNCT. We evaluate the dose of BNCT treatment in the thoracic region, especially the lungs. BNCT has predominantly been applied to the head and neck region; thus, this study is significant for BNCT in the thoracic region.

We present the pulmonary CT findings from the first three cases after BNCT for breast cancer, highlighting the potential and safety of BNCT in thoracic applications.

## Case presentation

Clinical study overview

This study (jRCTs031220371) utilizes CICS-2 manufactured by the CICS as a neutron irradiation system, and Borofalan(^10^B) produced by Stella Pharma Corporation (Osaka, Japan). This study is a single-center, open-label study.

The BNCT treatment protocol was modified from the protocol developed by Kyoto University, Japan [4]. Borofalan (^10^B) was administered for a total of three hours. For the first two hours, the infusion rate was 200 mg/kg/h, and the infusion rate was set to 100 mg/kg/h for the remaining hour. Neutron irradiation was carried out during the last hour of administering Borofalan (^10^B) at a rate of 100 mg/kg/h. We set up the patient’s body as the target was close to the irradiation aperture.

The dose constraints that we employed were the maximum skin dose of 18 Gy-Eq, and the average dose of 5 Gy-Eq for both liver and lungs. The total equivalent dose (ED) was calculated by the factors and equation shown in Table 1. Additionally, the boron concentration ratios of skin to blood, normal tissue to blood, and tumor to blood were 1.4, 1.0, and 3.5, respectively.

**Table 1:** How to calculate the total equivalent dose. CBE: Compound Biological Effectiveness; RBE (Relative Biological Effectiveness; RBE_N_, RBE_H_, and RBE_G_ are RBE values for nitrogen, hydrogen, and gamma-ray, respectively. Equivalent dose (Gy-Eq)=CBE×Boron dose+RBE_N_­×Nitrogen dose+RBE_H_×Hydrogen dose+RBE_G_×Gamma ray dose

CBE/RBE factors	Tumor	Skin	Lung	Liver
CBE	4.0	2.5	2.3	4.25
RBE_N_	3.0	3.0	3.0	3.0
RBE_H_	3.0	3.0	3.0	3.0
RBE_G_	1.0	1.0	1.0	1.0

The protocol included blood tests and physical examinations to evaluate for acute toxicities at intervals of 1, 3, 7, 30, 60, and 90 days after BNCT. CT scans, MRI, or visual palpation was performed to evaluate the tumor in 1, 7, 30, 60, and 90 days. We examined the lungs using CT or MRI imaging techniques for the first three cases that did not have lung metastases before BNCT.

Case 1: 72-year-old woman

**Figure 1:**
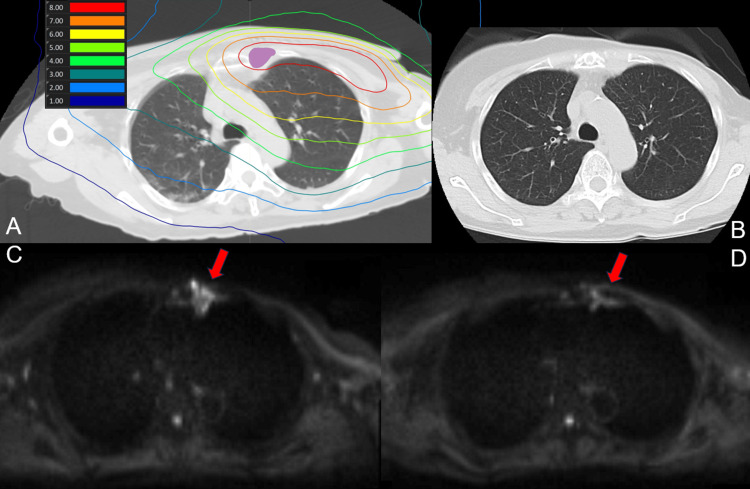
(A) Dose distribution map for BNCT (lung). Purple represents the GTV. (B) CT scan 90 days post-treatment shows no signs of radiation pneumonitis. (C) Pre-treatment diffusion-weighted imaging MRI. (D) MRI scan 90 days shows a decrease in signal intensity.

The patient received chemotherapy for left breast cancer 12 and a half years before BNCT treatment, followed by mastectomy (ypT2N1M0). A recurrence was later observed in the left parasternal lymph nodes, which was treated with hormone therapy and chemotherapy. The patient underwent irradiation of the left chest wall and parasternal area with 59 Gy delivered in 30 fractions four years ago. Although there were no subsequent recurrences, a relapse was noted in the left internal mammary lymph nodes, for which BNCT was administered. The prescribed dose was 31.4 Gy-Eq at minimum to gross tumor volume (GTV), and the average dose for the ipsilateral lung was 5 Gy-Eq. MRIs conducted one day and seven days after BNCT, and CT scans performed 30 and 90 days post-BNCT, showed no evidence of radiation pneumonitis. Figure 1 shows the dose distribution of the pulmonary region and the CT and MRI scans 90 days post-treatment.

Case 2: 61-year-old woman

**Figure 2:**
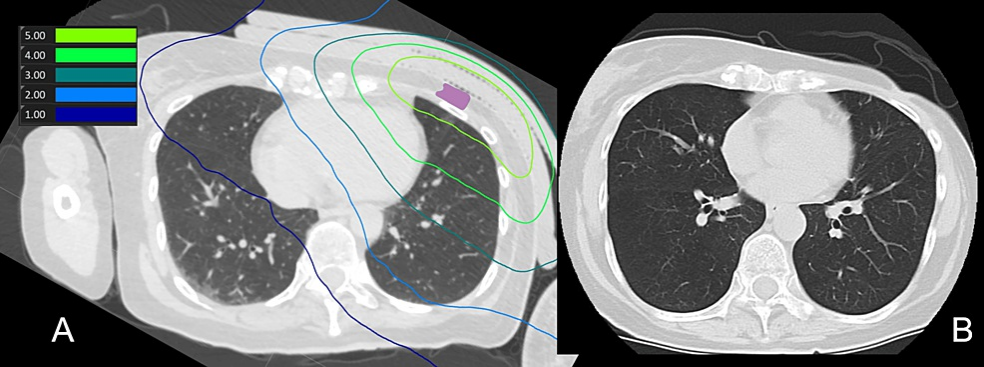
(A) Dose distribution map for BNCT (lung). Purple represents the GTV. (B) CT scan 90 days post-treatment, with no signs of radiation pneumonitis observed. The GTV is shrunk. BNCT: boron neutron capture therapy, GTV: gross tumor volume

Chemotherapy was prescribed for left breast cancer six and a half years before BNCT, followed by a mastectomy (ypT2N1M0), and the left chest wall was irradiated with 54 Gy in 30 fractions. Subsequently, a recurrence in the left chest was treated by surgery and radiotherapy with 50.4 Gy in 28 fractions three years after the first treatment, along with chemotherapy. A further recurrence on the left chest wall was observed, and BNCT was performed. The prescribed dose was 23.6 Gy-Eq at minimum, and the average dose was 2.7 Gy-Eq on the ipsilateral lung. CT scans conducted 1, 3, 7, 30, 60, and 90 days after BNCT showed no evidence of radiation pneumonitis. Figure 2 shows the pulmonary dose distribution, and the CT scan 90 days post-treatment.

Case 3: 52-year-old woman

The patient underwent chemotherapy for right breast cancer, followed by breast-conserving surgery and subsequent breast-conserving irradiation of 50 Gy in 25 fractions 10 years before BNCT. The patient had a local recurrence treated with surgery (rpT3N2M0) and chemotherapy three and a half years ago. Despite two more surgical interventions for local recurrences and hormone therapy, the cancer recurred, leading to the administration of BNCT.

**Figure 3:**
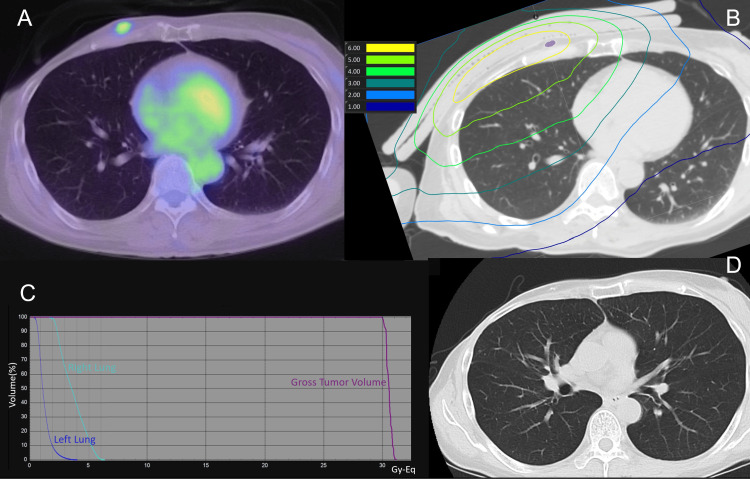
(A) Pre-treatment PET-CT. (B) Dose distribution map for BNCT (lung). Purple represents the GTV. (C) Dose-volume histogram. (D) CT scan 90 days post-treatment, showing a reduction in the GTV. BNCT: boron neutron capture therapy, GTV: gross tumor volume

The prescribed dose was 30 Gy-Eq at minimum, and the average dose for the ipsilateral lung was 3.7 Gy-Eq. CT scans conducted 1, 7, 30, 60, and 90 days after BNCT showed no evidence of radiation pneumonitis. Figure 3 shows the pulmonary dose distribution and the CT scan 90 days post-treatment.

In all cases, no grade 3 or higher adverse effects were observed within 90 days.

## Discussion

BNCT has been principally performed for head and neck cancers using RB-BNCT and AB-BNCT. Recent explorations have sought to expand BNCT’s applicability to additional malignancies. Suzuki et al. investigated the feasibility of BNCT for malignant pleural mesothelioma with RB-BNCT at the Institute for Integrated Radiation and Nuclear Science, Kyoto University [5]. Inoue et al. reported on RB-BNCT for a malignant peripheral nerve sheath tumor located in the right supraclavicular fossa, observing no adverse effects apart from temporary stomatitis [6]. In 2020, BNCT using a reactor was carried out at Osaka Medical College Hospital for a single patient with recurrent breast cancer following radiotherapy, with a reduction observed at four months and an increase at nine months. In this case, no side effects were reported other than a CTCAE grade 1 loss of appetite [7].

In this study, the constraint dose of lungs is 5 Gy-Eq on average, which was chosen from the calculation for AB-BNCT [8]. For case 1, the dose of 5 Gy-Eq on average was given to the left lung, and V_5Gy_ was 27.0%. The large region of the left lung received over 5 Gy-Eq; however, there was no sign of radiation pneumonitis, including cases 2 and 3 in the period of follow-up. The maximum, average, and minimum doses for the lungs were summarized in Table 2.

**Table 2:** The doses for the lungs.

	Ipsilateral lung			Contralateral lung		
	D_max_ [Gy-Eq]	D_ave_ [Gy-Eq]	D_min_ [Gy-Eq]	D_max_ [Gy-Eq]	D_ave_ [Gy-Eq]	D_min_ [Gy-Eq]
Case 1	8.9	5.0	0.9	5.9	2.4	0.4
Case 2	6.4	2.7	1.2	2.2	0.9	0.4
Case 3	6.3	3.7	1.5	4.1	1.3	0.4

In the current report, there were no findings of radiation pneumonitis within 90 days. It is known that, with conventionally fractionated radiotherapy using X-rays, a faint shadow corresponding to the irradiation field typically appears after a latency period of one to three months post irradiation. Given that conventional radiotherapy is administered over one to two months, the relatively short observation period in this report could be considered a limitation.

## Conclusions

BNCT has been investigated rigorously to expand additional malignancies. It is well-known that BNCT can be applied to breast cancer, and we performed BNCT for patients with recurrent breast cancer after radiation therapy. This study is a significant milestone as the first application of AB-BNCT for breast cancer treatment. The average dose of the ipsilateral lung was 5 Gy-Eq at maximum in this study. In all cases, there was no incidence of radiation pneumonitis within 90 days. While long-term observation is still necessary, these findings indicate the potential applicability of BNCT for the treatment of thoracic regions.
